# Comprehensive genetic analysis of histological components of combined small cell carcinoma

**DOI:** 10.1111/1759-7714.14574

**Published:** 2022-07-11

**Authors:** Yuko Iida, Yoko Nakanishi, Tetsuo Shimizu, Masayuki Nomoto, Yoshiko Nakagawa, Reiko Ito, Noriaki Takahashi, Shinobu Masuda, Yasuhiro Gon

**Affiliations:** ^1^ Division of Respiratory Medicine, Department of Internal Medicine Nihon University School of Medicine Tokyo Japan; ^2^ Division of Oncologic Pathology, Department of Pathology and Microbiology Nihon University School of Medicine Tokyo Japan

**Keywords:** achaete‐scute homolog‐1, heterogeneity, next‐generation sequencing, small‐cell lung cancer, somatic mutations

## Abstract

**Background:**

Combined small‐cell lung cancer (cSCLC) is a rare type of small‐cell lung cancer (SCLC) that includes both SCLC and non‐small‐cell lung cancer (NSCLC). The molecular biological mechanisms underlying the heterogeneity of histological types in combined or metachronously transformed SCLC (mtSCLC) remain unclear. This study aimed to investigate the relationship between genetic alterations and each histological component heterogeneously detected in cSCLC and mtSCLC.

**Methods:**

This study included four cSCLC cases and one mtSCLC case. Formalin‐fixed and paraffin‐embedded sections of each histological component of these tumors were subjected to next‐generation sequencing (NGS) and quantitative reverse transcription‐polymerase chain reaction to investigate the genetic mutations and expression levels of neuroendocrine cell‐specific transcription factors (achaete‐scute homolog‐1 [ASCL1], brain‐2 [BRN2] also known as POU domain class 3 transcription factor 2, nuclear factor 1 B [NF1B], insulinoma‐associated protein 1 [INSM1], and thyroid transcription factor‐1 [TTF‐1]).

**Results:**

NGS analysis revealed that SCLC and NSCLC components share the same somatic mutations detected most frequently in *TP53*, and also in *RB1* and *EGFR*. Gene expression analysis showed *ASCL1* expression was significantly lower in the NSCLC component than in the SCLC component.

**Conclusion:**

We conclude that the morphological evolution of heterogeneous histological components in cSCLC may be associated with differences in *ASCL1* expression levels, but not in acquired somatic gene mutations.

## Graphical abstract

We investigated the relationship between genetic alterations and each histological component heterogeneously detected in combined SCLC (cSCLC) and metachronously transformed SCLC. The different histological components of cSCLC shared the same major somatic mutations, but *ASCL1* expression was lower in the NSCLC component than in the SCLC component. Each histological component of cSCLC undergoes morphological evolution depending on the difference in *ASCL1* expression, not due to the differences in acquired somatic mutations.

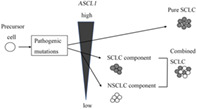

## INTRODUCTION

Small‐cell lung cancer (SCLC) is a highly aggressive neuroendocrine tumor that accounts for 13% of all lung cancers worldwide.^1^ Combined SCLC (cSCLC) is a type of SCLC with an incidence of 28% of all SCLC cases diagnosed by surgical specimens.^2^ The World Health Organization (WHO) Tumor Classification defines it as SCLC combined with additional components of any histological type of non‐small‐cell lung carcinoma (NSCLC).^3^ Cases of NSCLC have been described with epidermal growth factor receptor (EGFR) gene mutation that was metachronously transformed to SCLC after treatment with EGFR‐tyrosine kinase inhibitors (TKIs).^4^, ^5^ However, the cell origin of SCLC and NSCLC is usually thought to be different: SCLC generally arises from neuroendocrine cells or neuroendocrine progenitors, whereas adenocarcinoma originates from alveolar type 2 and club cells.^6^, ^7^ Another report indicated that alveolar type II cells might form SCLC, although less frequently than neuroendocrine cells.^8^ The cell origin of cSCLC and metachronously transformed SCLC (mtSCLC) underlying the heterogeneity of different histological components is unknown.

The genetic background of the two histological components of cSCLC is unclear. Only a few reports have investigated the genetic profiles of different histologic components in cSCLC using next‐generation sequencing (NGS).^9^, ^10^

Achaete‐scute homolog‐1 (ASCL1), a basic helix–loop–helix transcription factor, is necessary to initiate the development of SCLC in a mouse model and induce neuroendocrine differentiation in SCLC.^11^, ^12^, ^13^ The transcription factor nuclear factor 1 B (NF1B) is targeted by ASCL1 and drives tumor initiation and progression in mouse models of SCLC.^14^, ^15^ Insulinoma‐associated protein 1 (INSM1) is a zinc‐finger transcription factor. It is a crucial regulator of ASCL1, brain‐2 (BRN2), and neuroendocrine molecules in lung cancer cells and plays a role in the proliferation and apoptosis of SCLC.^16^ Thyroid transcription factor‐1 (TTF‐1) expression levels have been associated with neuroendocrine differentiation via the expression of its regulators, such as ASCL1 and NF1B, in SCLC.^17^ The roles of these transcription factors in the histological differences in cSCLC and mtSCLC have not been elucidated. This study aimed to clarify the status of genetic mutations and gene expressions related to morphological heterogeneity in cSCLC and mtSCLC.

## METHODS

### Patients

Patients with cSCLC, mtSCLC, and pure SCLC consecutively diagnosed and treated at the Nihon University Itabashi Hospital (Tokyo, Japan) between 2010 and 2019 were enrolled in this study. The study design was approved by the Institutional Review Board (265‐0, 30‐14‐0) according to the Declaration of Helsinki. The study investigated somatic mutations in targeted cancer panels; germline mutations were excluded.

Patients' clinical information was extracted from the medical records at Nihon University Itabashi Hospital (Table 1). Diagnoses of cSCLC, mtSCLC, and pure SCLC were based on the 2021 WHO classification of lung tumors^3^ by trained histopathologists. Four patients (cases 1–4) had limited cSCLC. One patient (case 5) had advanced mtSCLC that had progressed from adenocarcinoma. The FFPE tissues of primary and/or metastatic lesions of these patients were subjected to immunohistochemical analysis. Three cSCLC samples (cases 1–3) and one mtSCLC sample (case 5) were subjected to NGS analysis, and four cSCLC samples (cases 1–4) and six pure SCLC samples (Table S1) were subjected to quantitative RT‐PCR analyses.

**TABLE 1:** Clinicopathological features of patients with combined SCLC and metachronously transformed SCLC

Cases	Synchronous/metachronous	Histological component	Stage	Sex	Age	Smoking	Treatment	Follow‐up
1	Synchronous	SC/Ad	IA	F	76	+	Surgery	Died 20 months after diagnosis
							Adjuvant chemotherapy	
2	Synchronous	SC/Sq	IIB	M	81	+	Surgery	Transferred 6 months after diagnosis
								
3	Synchronous	SC/Ad	IB	M	60	+	Surgery	Alive 38 months after diagnosis
							Adjuvant chemotherapy	
4	Synchronous	SC/Sq	IIIB	M	76	+	surgery	No information
							Adjuvant chemotherapy	
5	Metachronous	SC/Ad	IV	F	70	−	EGFR‐TKI (before SC)	Transferred 73 months after diagnosis
							Chemotherapy (after SC)	

*Abbreviations*: Ad, adenocarcinoma; EGFR‐TKI, epidermal growth factor receptor‐tyrosine kinase inhibitor; SC, small‐cell lung cancer; Sq, squamous cell carcinoma.

### Immunohistochemistry

The expressions of CD56, synaptophysin, chromogranin A, ASCL1, INSM1, TTF‐1, and Ki‐67 were evaluated using immunohistochemistry. The 4‐μm thick sections were mounted on silane‐coated glass slides. After deparaffinization, samples were boiled in citrate buffer (pH 6.0) for CD56, synaptophysin, and chromogranin A, and in ethylenediamine tetraacetic acid buffer (pH 9.0) for ASCL1, INSM1, and TTF‐1, as antigen retrieval. Subsequently, an automated staining system (Histostainer; Nichirei Bioscience, Tokyo, Japan) was used for immunostaining, which was performed as follows: (1) blocking with 3% hydrogen peroxide to eliminate endogenous peroxidase for 5 min at room temperature; (2) incubation with 1/50 primary mouse monoclonal anti‐Ki‐67 antibody (clone MIB‐1; Agilent Technologies Inc.), 1/50 mouse monoclonal anti‐CD56 antibody (clone 123‐C3, Agilent Technologies Inc.), 1/100 mouse monoclonal anti‐chromogranin A antibody (clone DAK‐A3, Agilent Technologies Inc.), 1/50 rabbit polyclonal anti‐synaptophysin antibody (Agilent Technologies Inc.), 1/1000 rabbit monoclonal anti‐ASCL1 antibody (clone EPR19592; Abcam PLC), 1/100 mouse monoclonal anti‐INSM1 antibody (clone A‐8; Santa Cruz Biotechnology Inc.), and 1/100 mouse monoclonal anti‐TTF‐1 antibody (clone 8G7G3/1; Agilent Technologies) each for 30 min at room temperature; (3) washing with phosphate buffered saline (PBS); (4) incubation with the polymer second antibody (Simple stain Max PO Multi; Nichirei Bioscience) for 30 min at room temperature; (5) washing with PBS; (6) dyeing with 3,3‐diaminobenzidine for 10 min at room temperature; (7) washing with PBS; (8) counterstaining with hematoxylin; and (9) dehydrating and covering with cover glasses. The detailed methods have been previously reported.^17^

### Total DNA extraction, targeted enrichment, and sequencing

The 8‐μm thick sections were mounted on regular glass slides. After deparaffinization, the target tumor cells were macro‐dissected and collected in a 1.5‐mL tube. The target tumor cells were identified using hematoxylin–eosin (HE) staining. Genomic DNA was extracted using the QIAamp DNA FFPE Tissue Kit (Qiagen) according to the manufacturer's instructions. The concentration and quality of the extracted DNA were measured using the GeneRead DNA QuantiMIZE Kit (Qiagen) before the targeted enrichment. The coding regions and exon/intron junctions of 72 oncogenes were enriched by multiplex PCR using the QIAseq Targeted DNA Human Lung Cancer Panel (DHS‐005Z; Qiagen) according to the manufacturer's instructions and sequenced using NextSeq 500 (Illumina) in 151‐base pair (bp) paired‐end reads. The average read depth of coverage was set at 3400× to allow the detection of rare mutations and to accurately estimate variant allele frequencies.

### Alignment and somatic variant calling

Alignment and somatic variant calling were performed using the Qiagen Data Analysis Center web service (https://ngsdataanalysis.qiagen.com/QIAseqDNA). Original FASTQ files generated by NextSeq were uploaded to the web service. Subsequently, smCounter v2 was used for somatic variant calling.^18^

### Variant annotation and filtering

Functional annotations of the Ensembl database GRCh37.75^19^ and the possible effects of variants were added using SnpEff version 4.2.^20^ Using these annotations, variants were filtered first for those that were predicted to alter amino acid sequences (missense, nonsense, and splice‐site mutations and indels in coding regions), then for those that were rare (<1.0% minor allele frequencies in the HapMap‐JPT [Japanese in Tokyo, Japan], the 1000 Genomes EAS [East Asian population including 104 Japanese individuals], or the Human Genetic Variation Database [http://www.genome.med.kyoto-u.ac.jp/SnpDB/]). Furthermore, we used the genome data of eight previously sequenced healthy Japanese men aged over 70 years with no relevant medical history for variant filtering. Those with a variant allele frequency (VAF) ≥10% were targeted. These mutations were validated and analyzed in normal lung tissues using Sanger sequencing.

### Total RNA extraction and cDNA synthesis

The 8‐μm thick sections were mounted on regular glass slides. After deparaffinization, the target tumor cells, identified using HE staining, were dissected and collected in 1.5‐mL tubes. Total RNA was extracted using an RNeasy FFPE Kit (Qiagen) according to the manufacturer's instructions. The RNA samples were dissolved in 5 μl of RNase‐free water, and the concentration was measured using the Nanodrop (Thermo Fisher Scientific Inc.). Total RNA samples were stored at −80°C until use. Genomic DNA was eliminated and cDNA was synthesized using the QuantiTect Reverse Transcription Kit (Qiagen) according to the manufacturer's instructions.

### Quantitative RT‐PCR assay

We investigated the mRNA expression levels of TTF‐1, ASCL1, BRN2, NF1B, INSM1, and the internal control, glyceraldehyde‐3‐phosphate dehydrogenase (GAPDH), using quantitative RT‐PCR. Quantitative RT‐PCR was performed using 1 μl of cDNA, TaqMan probes (Thermo Fisher Scientific Inc.), and a Step One Plus quantitative RT‐PCR machine (Thermo Fisher Scientific Inc.). The premixed primers and probes were as follows: ASCL1 (Assay ID: Hs04187546_g1), BRN2 (Assay ID: Hs00271595_s1), NF1B (Assay ID: Hs01029175_m1), TTF‐1 (Assay ID: Hs00968940_m1), INSM1 (Assay ID: Hs00357871_s1), and GAPDH (Assay ID: Hs99999905_m1). The quantitative RT‐PCR thermal cycling profile was as follows: 95°C for 10 min, followed by 50 cycles of 95°C for 15 s and 60°C for 1 min. The expression levels of the target mRNA were calculated using the ΔCt method, with GAPDH mRNA expression as the reference.^21^

### Statistical analysis

The correlations between the components of SCLC and NSCLC in cSCLC and mtSCLC observed by immunohistochemistry (with respect to the markers synaptophysin, chromogranin A, CD56, ASCL1, INSM1, and TTF‐1) were analyzed using Fisher's exact test. Correlations in terms of the Ki‐67 expression level between components of SCLC and NSCLC in cSCLC and mtSCLC were analyzed using the Mann–Whitney U test. The differences in mRNA expression levels among pure SCLC, SCLC component, and NSCLC component in cSCLC and mtSCLC were analyzed using the Kruskal–Wallis test. The Steel–Dwass post‐hoc test was used for multiple comparisons. All statistical analyses were performed using EZR (Saitama Medical Center, Jichi Medical University, Saitama, Japan), a graphical interface for R (The R Foundation for Statistical Computing).^22^

## RESULTS

### Immunohistochemical findings

**FIGURE 1:**
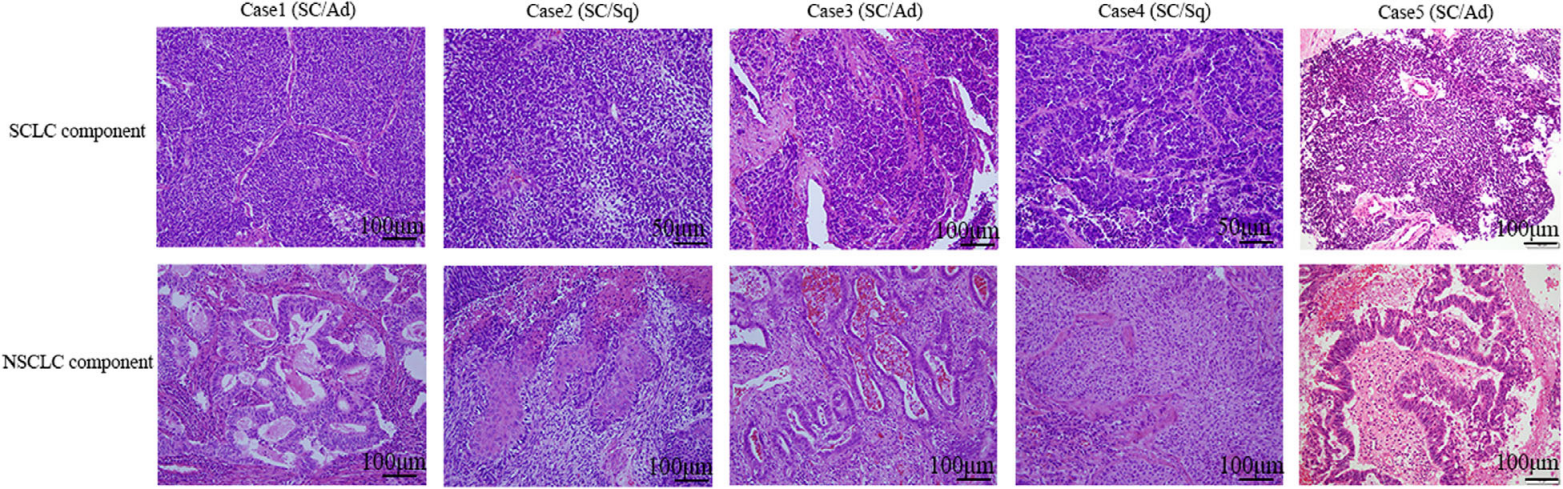
Histologic features of SCLC and NSCLC components in cSCLC and mtSCLC. Representative features of hematoxylin and eosin staining are presented. Scale bars of 50 μm and 100 μm are shown. SC, small‐cell lung cancer; Ad, adenocarcinoma; Sq, squamous cell carcinoma

Immunohistochemical neuroendocrine and proliferation markers were evaluated in the SCLC and NSCLC components (Figure 1). The SCLC component showed higher positive rates for synaptophysin, CD56, ASCL1, and INSM1, and a higher labeling index for Ki67 compared to the NSCLC component (Table 2 and Figure S1). The significant difference in the Ki‐67 labeling index (%) was higher in the SCLC component (mean 72%) than in the NSCLC component (mean 30%). There were no significant differences in chromogranin A or TTF‐1 expression (Table 2).

**TABLE 2:** Immunohistochemical features between different histological components in combined SCLC and metachronously transformed SCLC

Case	Histological component	Syn	Chromo	CD56	ASCL1	TTF‐1	INSM1	Ki 67 labeling index (%)
1	SC	+	−	+	−	+	−	90
	Ad	−	−	−	−	+	−	20
								
2	SC	+	−	+	±	+	±	80
	Sq	−	−	−	−	−	−	30
								
3	SC	+	+	+	+	++	+	80
	Ad	−	−	−	−	++	−	70
								
4	SC	++	++	++	++	−	++	30
	Sq	−	−	±	−	−	−	10
								
5	SC	++	−	++	+	++	++	80
	Ad	−	−	++	±	++	−	70
								
Total	SC	5/5**	2/5	5/5*	4/5*	4/5	4/5*	72 (30–90)*
	NSC	0/5	0/5	2/5	1/5	3/5	0/5	30 (10–70)

* *p* < 0.05.

** *p* < 0.01.

Total Ki67 labeling index is shown as mean and range.

*Abbreviations*: Ad, adenocarcinoma; Chromo, chromogranin A; NSC, non‐small‐cell carcinoma; SC, small‐cell carcinoma; Sq, squamous cell carcinoma; Syn, synaptophysin.

Immunohistochemical evaluations: −, negative; ±, rare; +, focal; ++, diffuse.

The Total column shows the positivity/total cases for each immunohistochemical antibody between small‐cell carcinoma and non‐small‐cell carcinoma.

### Genetic mutations in SCLC and NSCLC components

Genetic mutations in three cSCLC cases and one mtSCLC case were analyzed using samples with sufficient levels of DNA quality and volume. The tumor contents comprised 80–90% of the SCLC component and 20–80% of the NSCLC component. Table 3 lists the somatic mutations that were detected in each case. Figure S2 shows the somatic mutations validated using Sanger sequencing. In four cases, 17 mutations in nine genes (*EGFR*, *RB1*, *TP53, MUC16*, *SMARCA4*, *KDR*, *PKHD1*, *KMT2D*, and *RBM10*) were found. The most common mutation type was the missense mutation (88%, 15/17 mutations). According to the ClinVar database, two pathogenic somatic mutations were clinically significant: p.Ser768Ile in *EGFR* (case 1) and p.Arg251* in *RB1* (case 5), one pathogenic/likely pathogenic somatic mutation, p.Arg158His in *TP53* (case 1), and one likely pathogenic somatic mutation, p.Arg175Leu in *TP53* (case 5). A drug response variant was also detected (p.Leu858Arg in *EGFR* [case 5]). The somatic mutations with uncertain significance were p.Pro142Leu in *TP53* (case 5), p.Ala159Val in *TP53* (case 2), p.Lys1540Arg in *SMARCA4* (case 5), p.Val774Met in *EGFR* (case 1), and p.Glu654Lys in *PKHD1* (case 2).

**TABLE 3:** Genomic analysis in combined SCLC and metachronously transformed SCLC

Cases	Tumor content rate	Syn/Meta	Type	VAF	Gene	Mutation type	HGVSc	HGVSp	ClinVar interpretation	Sample no.^a^
1	SC: 90% Ad: 20%	Syn	SC	0.6505	*EGFR*	Missense	c.2303G > T	p.Ser768Ile	Pathogenic	1
			Ad	0.4727						2
			SC	0.6504	*EGFR*	Missense	c.2320G > A	p.Val774Met	Uncertain significance	3
			Ad	0.4741						4
			SC	0.7796	*TP53*	Missense	c.473G > A	p.Arg158His	Pathogenic/Likely pathogenic	5
			Ad	0.5872						6
2	SC:90% Sq: 80%	Syn	SC	0.6372	*KDR*	Missense	c.1292C > T	p.Pro431Leu		7
			Sq	0.6513						8
			SC	0.6406	*PKHD1*	Missense	c.1960G > A	p.Glu654Lys	Uncertain significance	9
			Sq	0.6868						10
			SC	0.6107	*PKHD1*	Missense	c.1139 T > C	p.Phe380Ser		11
			Sq	0.6531						12
			SC	0.4899	*KMT2D*	Splice donor/intron	c.16052 + 1G > A			13
			Sq	0.5891						14
			SC	0.8426	*TP53*	Missense	c.476C > T	p.Ala159Val	Uncertain significance	15
			Sq	0.8609						16
3	SC: 80% Ad: 50%	Syn	SC	0.6042	*RB1*	Missense/splice region	c.861G > T	p.Glu287Asp		17
			SC	0.5063	*MUC16*	missense	c.4539 T > G	p.Asp1513Glu		18
			Ad	0.4321						19
			SC	0.7765	*RBM10*	Missense	c.920 T > A	p.Leu307Gln		20
5	SC: 80% Ad: 70%	Meta	SC	0.8944	*EGFR*	Missense	c.2573 T > G	p.Leu858Arg	Drug response	21
			Ad	0.6461						22
			SC	0.9450	*RB1*	Nonsense	c.751C > T	p.Arg251*	Pathogenic	23
			Ad	0.7846						24
			SC	0.9353	*TP53*	Missense	c.524G > T	p.Arg175Leu	Likely pathogenic	25
			Ad	0.6989						26
			SC	0.9595	*TP53*	Missense	c.425C > T	p.Pro142Leu	Uncertain significance	27
			Ad	0.7290						28
			Ad	0.3437	*MUC16*	Missense	c.37181 T > A	p.Phe12394Tyr		29
			SC	0.6614	*SMARCA4*	Missense	c.4619A > G	p.Lys1540Arg	Uncertain significance	30
			Ad	0.6537						31

Abbreviations: Ad, adenocarcinoma; HGVS, Human Genome Variation Society; Meta, metachronous; SC, small‐cell lung cancer; Sq, squamous cell carcinoma; Syn, synchronous.

^a^ Sample no. corresponds to Figure S1.

In each case, most genetic mutations were shared between SCLC and NSCLC components: 3/3 (100%) in case 1, 5/5 (100%) in case 2, 1/3 (33%) in case 3, and 5/6 (83%) in case 5. The common shared genetic mutations were *TP53* (3/4 cases), *EGFR* (2/4 cases), *KDR*, *PKHD1*, *KMT2D*, *MUC16*, *RB1*, and *SMARCA4* (1/4 cases).

### mRNA expression levels of *ASCL1* , *BRN2* , *NF1B* , *TTF‐1*, and *INSM1* in the SCLC and NSCLC components of cSCLC and pure SCLC

**FIGURE 2:**
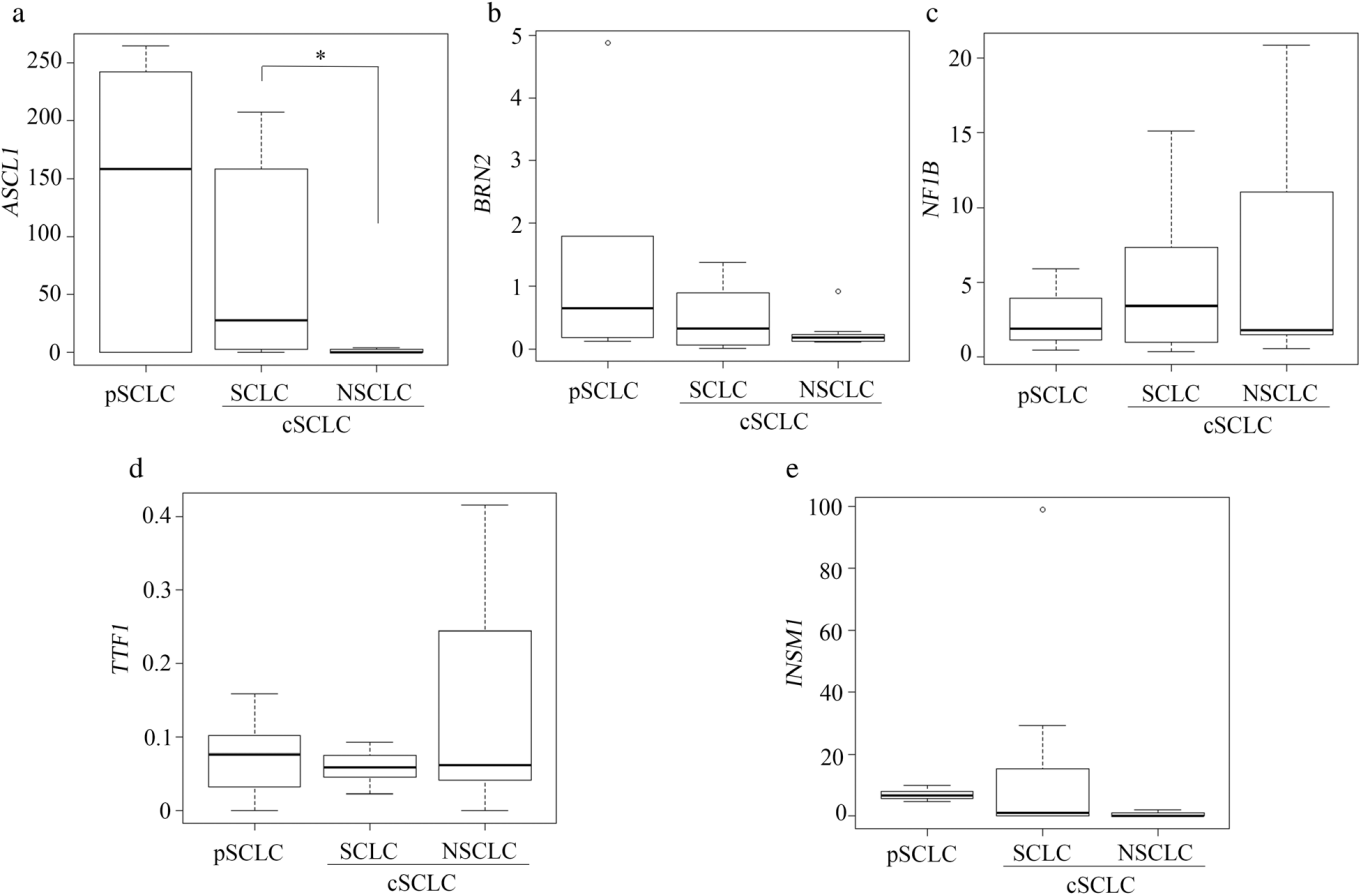
mRNA expression of neuroendocrine‐related genes of pure SCLC and SCLC and NSCLC component in cSCLC mRNA expression normalized to GAPDH was compared to that of *ASCL1* (a), *BRN2* (b), *NF1B* (c), *TTF‐1* (d), and *INSM1* (e). The results were statistically analyzed using the Mann–Whitney U test. Bars represent mean ± standard deviation. *p < 0.05. NS, not significant

The mRNA expression levels of *ASCL1* were significantly lower in the NSCLC component than in the SCLC component of cSCLCs (*p* = 0.029; Figure 2a). The *ASCL1* expression level tended to be higher in the pure SCLC than in SCLC components of cSCLC, although this was not statistically significant (Figure 2a). *BRN2*, *NF1B*, *TTF‐1*, and *INSM1* expression levels did not differ significantly between the pure SCLC and SCLC components or between the SCLC and NSCLC components of cSCLC (Figure 2b–e).

## DISCUSSION

The present study demonstrated that cSCLC and mtSCLC share the same major gene mutations in the SCLC and NSCLC components. However, in the NSCLC component, the *ASCL1* expression was significantly lower than in the SCLC component of the cSCLC.

Similar to our result, a previous study also showed that approximately 75% of the identified somatic mutations, such as *TP53*, were present in both components of three cSCLC cases.^9^ These results suggest that SCLC and NSCLC components in cSCLC originate from a common ancestor because they have a similar genetic background. In this study, the most common gene mutation was the *TP53* mutation found in all three cases. The *TP53* mutations have been reported in approximately 90% of SCLC cases and 46% of NSCLC (adenocarcinoma) cases.^23^, ^24^ Furthermore, shared *RB1* mutations were observed in one case. *RB1* mutations have been reported in approximately 65% of SCLC cases and 4% of NSCLC (adenocarcinoma) cases.^23^, ^24^ Bi‐allele *TP53* and *RB1* mutations are early and necessary key events in the development of pure SCLC in humans.^14^, ^25^ The same *TP53* and *RB1* mutations were found in both SCLC and NSCLC histological components in this study. From the results of these gene mutations, the cell origin of each histological component of cSCLC may be closer to that of pure SCLC than that of NSCLC. In addition, pathogenic *EGFR* mutations were shared by both SCLC and NSCLC. Previous reports have shown that different histological components harbor the same EGFR mutation in transformation into SCLC as a mechanism of resistance to EGFR TKIs.^5^, ^26^ In this study, one cSCLC case had an EGFR mutation in both histological components, independent of transformation into SCLC as a mechanism of resistance to EGFR TKIs. Since there have been previous reports that cSCLC responds to EGFR‐TKI, it may be important to perform an EGFR gene mutation analysis in cSCLC to obtain additional therapeutic options.^26^

**FIGURE 3:**
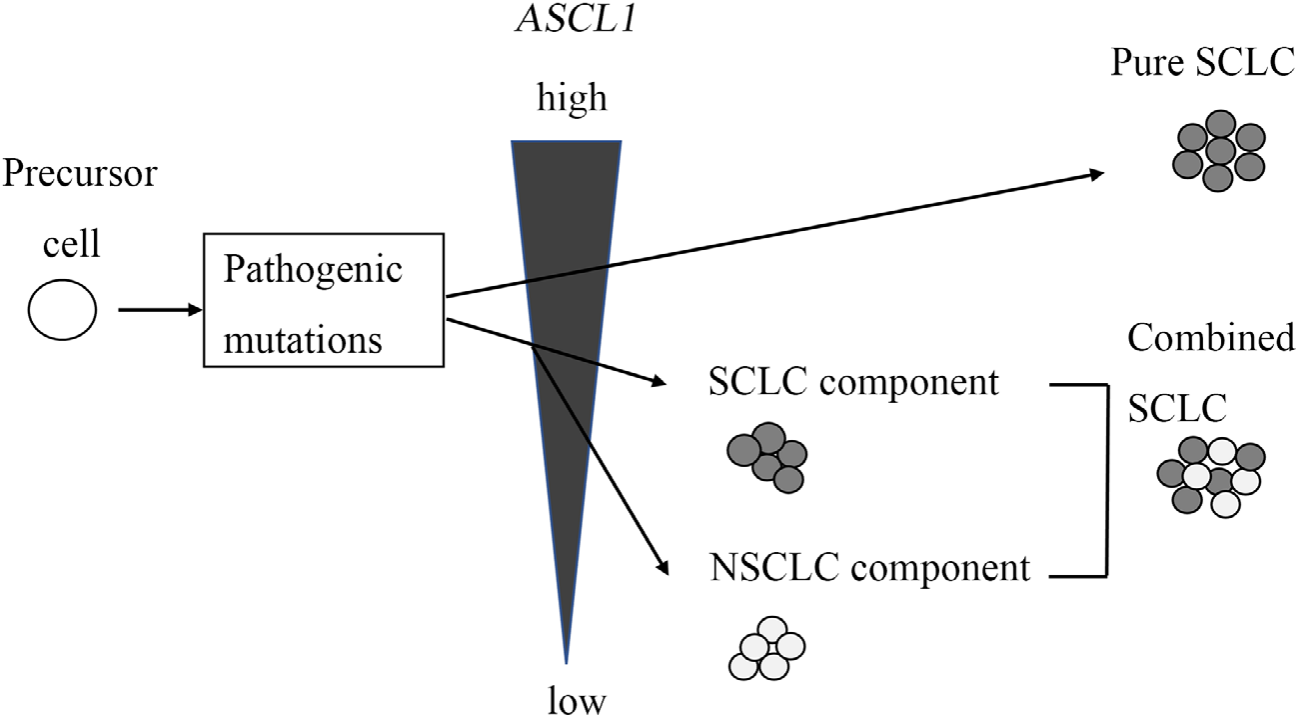
A hypothetical schematic representation of the carcinogenesis pathway in pure SCLC and cSCLC due to different levels of *ASCL1* expression

ASCL1 regulates neuroendocrine differentiation; in particular, it upregulates synaptophysin and contributes to proliferation and migration by targeting cyclin‐dependent kinase 5 in SCLC.^27^, ^28^, ^29^, ^30^ In the present study, the more frequent immunohistochemical positivity for synaptophysin, CD56, ASCL1, and INSM1 and a higher labeling index for Ki67 in the SCLC were correlated with higher *ASCL1* expression. Our results support the previous reports and suggest that ASCL1 plays an important role in the development of SCLC. Interestingly, *ASCL1* expression tended to be highest in pure SCLC and was lower in the NSCLC component than in the SCLC component. Cases that did not express ASCL1 and NEUROD1 in cSCLC compared to pure SCLC have been described.^31^ Decreasing ASCL1 expression in the NSCLC component is considered to be associated with the activation of NOTCH signaling regulated by histone modification and differentiation to NSCLC from SCLC.^32^ Another report hypothesized that cSCLC might originate from pure SCLC, partially decreasing ASCL1 in the NSCLC component.^33^ Our results suggest that morphological transformation into NSCLC from SCLC might occur depending on decreased *ASCL1* expression, but not because of genetic mutations, because of the presence of highly similar genetic backgrounds between the SCLC and NSCLC components (Figure 3).

In the present series, there was a peculiar case with lower *ASCL1* expression in both SCLC and NSCLC components (case 1). SCLC subtypes are recently defined by differential expression of four key transcription regulators: ASCL1, NEUROD1, yes‐associated protein 1 (YAP1) and POU2F3.^34^ SCLCs with low expression of neuroendocrine markers are classified as YAP1 or POU2F3 types. Whether the cSCLC of case1 represents the YAP1 or POU2F3 type remains to be addressed. SCLC subtyping is more important clinically because the therapeutic potential of inhibitors targeting delta‐like protein 3 (DLL3), an actionable target of ASCL1, is currently under further investigation in clinical trials.^35^ In ASCL1‐high SCLCs, DLL3‐targeting therapy is predicted to be more effective owing to the higher expression of DLL3.^36^ Our results suggest that targeted therapy may be less effective in cSCLC than in pure SCLC because of lower levels of *ASCL1*. Further clinical investigations of cSCLC are necessary.

This study has some limitations. First, the sample size was small. The small sample size might make it difficult to clarify the expression levels of *NF1B*, *BRN2*, *TTF‐1*, and *INSM1*. Second, the sensitivity of the methodologies may have influenced the results. The genetic mutations found in NGS were validated using Sanger sequencing, which has lower sensitivity than NGS, therefore gene mutations that could not be verified by Sanger sequencing could not be identified. Third, germline mutations were not analyzed in the present study. One mutation (case 3, MUC16) was found in cancerous and noncancerous lung tissues as a secondary finding, which might have resulted from a mixture of cancerous tissues or a germline mutation; however, the ClinVar database has no information regarding this mutation. Thus, this mutation warrants further investigation. Finally, a small number of different histological components might have been admixed. However, each component was collected separately as much as possible via macrodissection.

In conclusion, this study demonstrated that each histological component in SCLC may have morphological evolution depending on the difference in *ASCL1* expression, not due to the differences in acquired somatic mutations.

## DISCLOSURE

The authors have no conflicts of interest to declare.

## FUNDING

This work was supported by JSPS KAKENHI (Grant Number: 19 K17651).

## Supporting information


**Supporting Information Figure S1** Representative images in the immunohistochemical expression of synaptophysin, chromogranin A, CD56, ASCL1, TTF‐1, INSM1, and Ki67 in a pure SCLC case (case 3) and the SCLC and NSCLC components of a cSCLC case (case 3). Each bar shows 50 μm

**Supporting Information Figure S2** The arrangement with Sangers sequencing in each case Mutations in the SCLC component (upper line) and NSCLC (middle line) and the arrangement of the same place in normal lung tissue (bottom line). Black arrows indicate mutated bases
