# Supplementary figures and images for: Comprehensive genetic analysis of histological components of combined small cell carcinoma
Source: Thorac Cancer. 2022 Jul 11;13(16):2362–70. doi: 10.1111/1759-7714.14574 (PMC9376179; doi:10.1111/1759-7714.14574)

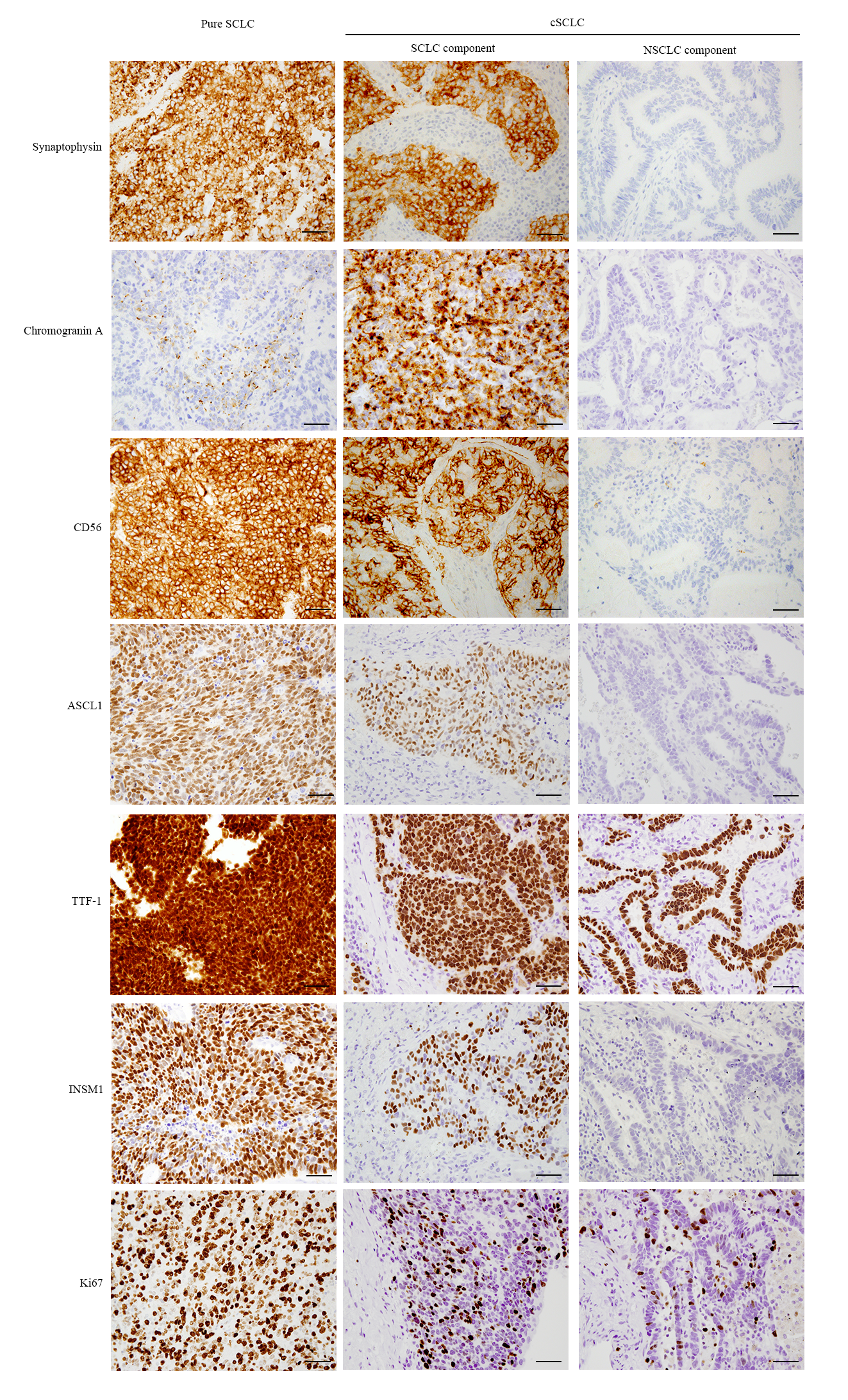

Supplement: Supplementary file 1 — Supporting Information Figure S1 Representative images in the immunohistochemical expression of synaptophysin, chromogranin A, CD56, ASCL1, TTF‐1, INSM1, and Ki67 in a pure SCLC case (case 3) and the SCLC and NSCLC components of a cSCLC case (case 3). Each bar shows 50 μm [file TCA-13-2362-s002.tif]

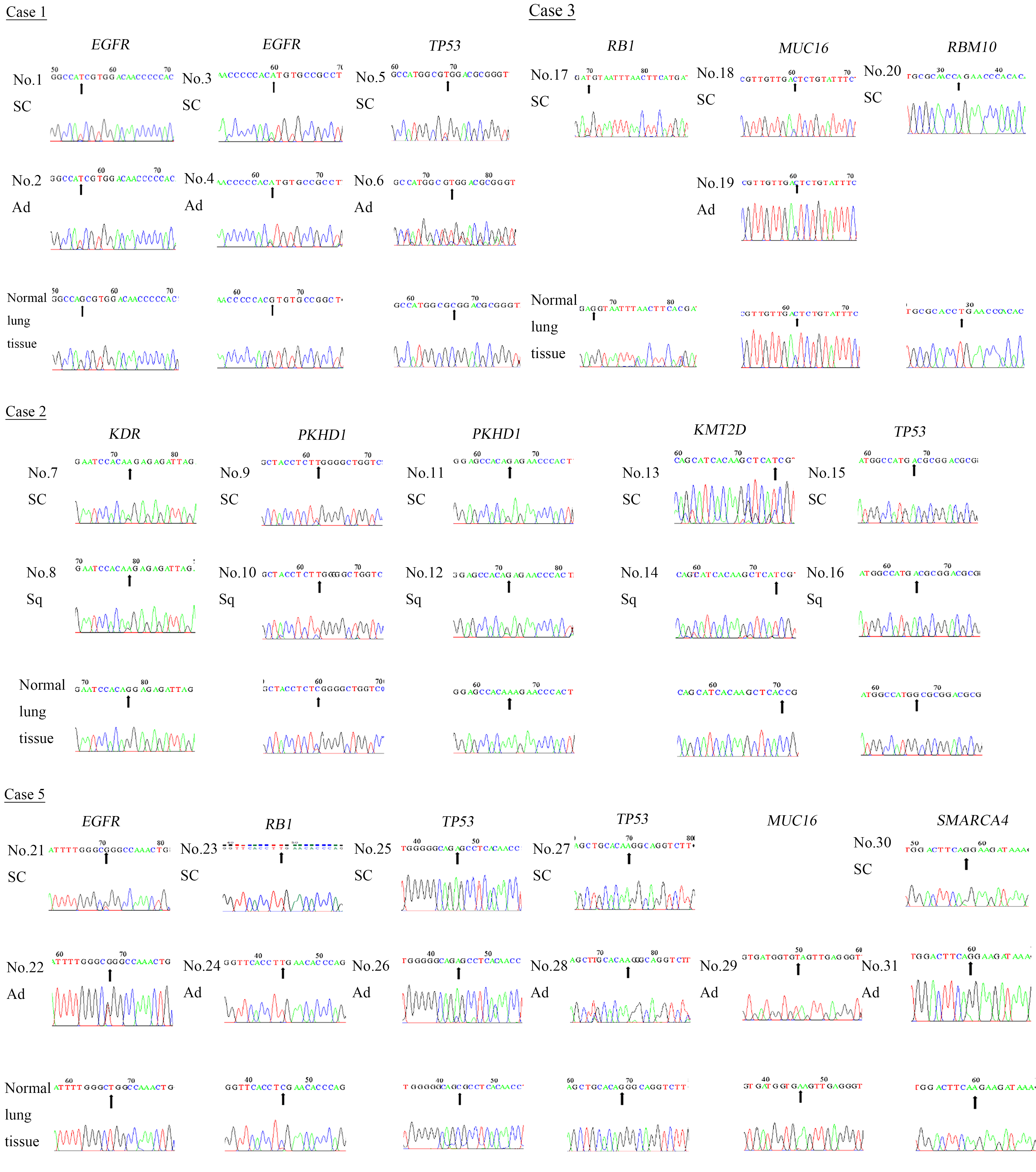

Supplement: Supplementary file 2 — Supporting Information Figure S2 The arrangement with Sangers sequencing in each case Mutations in the SCLC component (upper line) and NSCLC (middle line) and the arrangement of the same place in normal lung tissue (bottom line). Black arrows indicate mutated bases [file TCA-13-2362-s001.tif]
